# Hysterectomy at a Canadian tertiary care facility: results of a one year retrospective review

**DOI:** 10.1186/1472-6874-4-10

**Published:** 2004-11-23

**Authors:** Alina Toma, Wilma M Hopman, R Hugh Gorwill

**Affiliations:** 1 Department of Obstetrics and Gynaecology, Queen's University, Victory 4, Stuart Street, Kingston, Ontario, Canada K7L 2V7; 2 Clinical Research Centre, Kingston General Hospital and the Department of Community Health and Epidemiology, Queen's University, Kingston, Ontario, Canada K7L 2V7

## Abstract

**Background:**

The purpose of this study was to investigate the indications for and approach to hysterectomy at Kingston General Hospital (KGH), a teaching hospital affiliated with Queen's University at Kingston, Ontario. In particular, in light of current literature and government standards suggesting the superiority of vaginal versus abdominal approaches and a high number of concurrent oophorectomies, the aim was to examine the circumstances in which concurrent oophorectomies were performed and to compare abdominal and vaginal hysterectomy outcomes.

**Methods:**

A retrospective chart audit of 372 consecutive hysterectomies performed in 2001 was completed. Data regarding patient characteristics, process of care and outcomes were collected. Data were analyzed using descriptive statistics, t-tests and linear and logistic regression.

**Results:**

Average age was 48.5 years, mean body mass index (BMI) was 28.6, the mean length of stay (LOS) was 5.2 days using an abdominal approach and 3.0 days using a vaginal approach without laparoscopy. 14% of hysterectomies were performed vaginally, 5.9% were laparoscopically assisted vaginal hysterectomies and the rest were abdominal hysterectomies. The most common indication was dysfunctional or abnormal uterine bleeding (37%). The average age of those that had an oophorectomy (removal of both ovaries) was 50.8 years versus 44.3 years for those that did not (p < .05). Factors associated with LOS included surgical approach, age and the number of concurrent procedures.

**Conclusions:**

A significant reduction in LOS was found using the vaginal approach. Both the patient and the health care system may benefit from the tendency towards an increased use of vaginal hysterectomies. The audit process demonstrated the usefulness of an on-going review mechanism to examine trends associated with common surgical procedures.

## Background

In Canada in 2001, 446 hysterectomies were performed per 100 000 women [1]. The rate however varies considerably as a consequence of factors such as acceptability of medical management in areas where there is limited availability of gynaecologists [2] and a lack of dissemination and implementation of guidelines to direct treatment decisions [3].

In response to the consistent demand for this procedure, recent reports have identified hysterectomy as a key health care indicator used to measure and compare hospital performance. In particular, the Ontario Hospital Association has identified the ratio of vaginal (VH) to abdominal hysterectomy (AH) as a measure of hospital performance [4], with a more favorable grade awarded to those hospitals with a higher proportion of VHs. In addition, length of stay (LOS) and complication rates associated with hysterectomy are also used to grade hospital performance [4].

Considerable attention has also been directed towards the high rate of concurrent oophorectomy (removal of both ovaries) with this procedure. This rate is of particular concern in premenopausal women because of the early menopause that ensues.

The purpose of this study was to compare abdominal and vaginal approaches to hysterectomy, investigate the rate of concurrent oophorectomy, and identify factors associated with length of surgery, LOS and approach, by auditing all hysterectomies performed over a one-year period at a university teaching hospital.

## Methods

The study involved all patients who underwent a hysterectomy in 2001 at Kingston General Hospital (KGH), a teaching hospital affiliated with Queen's University at Kingston, Ontario. The Queen's University Health Sciences and Affiliated Teaching Hospitals Research Ethics Board approved the study (OBGY-117-03). There were no exclusion criteria. Patients were identified by medical record tracking using ICD-9 codes and charts were reviewed to collect patient characteristics, length of stay, length of surgery, indication for surgery and approach. Readmissions, complications, infections and repeat laparotomies were also assessed.

Menopause was defined as one year since the last menstrual period. Up to three indications for surgery were obtained from the chart, including those identified in clinic letters, admission sheets and operative reports. All indications were collected regardless of whether or not the post-operative diagnosis coincided with the preoperative diagnosis.

VH included laparoscopically assisted vaginal hysterectomy (LAVH) and AH included VH converted to AH unless otherwise noted. Readmission was defined as a visit to the emergency room or an admission to the same hospital with a diagnosis that was related (readmission to another facility was unlikely as KGH is the only tertiary care facility in the region). Post-operative infections were defined as those that occurred within 30 days of surgery. A complication of excessive bleeding was defined as an intra-operative hemorrhage requiring transfusion or laparotomy, post-operative hematoma/seroma formation, or a significant post-operative vaginal bleed that required medical attention. All complications that occurred during the surgery or within 30 days of surgery were recorded, other than problems associated with removal of catheter, urinary retention, hypertension, hypotension, pain control, nausea and vomiting or headache. Any repeat laparotomy or unplanned laparotomy (other than for conversion of VH to AH) that occurred during the surgery or within 30 days of discharge was also noted.

Follow up information was tracked using hospital chart and clinic note information from the six-week post-operative visit. All data were analyzed using SPSS statistical software (Version 11.0.1, SPSS Incorporated, Chicago, Illinois, 2002). Between-group comparisons utilized two-sample t-tests and one-way analysis of variance (continuous data) and Chi-square analyses (categorical data). Predictors of LOS and length of surgery were identified using linear regression, while predictors of surgical approach were identified using logistic regression. Variables were offered into the models on the basis of the strength of the bivariate associations with the outcomes (p < 0.20).

## Results

Three hundred and seventy two women underwent a hysterectomy in 2001. The characteristics of these patients can be found in Table 1. Sixty-nine percent were premenopausal at the time of the surgery.

**Table 1:** Patient Characteristics

	N	Minimum	Maximum	Mean	Std. Deviation
Age (years)	372	27	87	48.5	11.5
Body Mass Index	357	16	79	28.6	7.3
Parity	365	0	9	2.1	1.5
Length of Stay (days)	372	1	62	4.7	4.4
Length of Surgery (minutes)	369	38	390	104.4	46.4

The majority of hysterectomies were AH (78%), 14% were VH, 5.9% were LAVH and 2.2% were VH converted to AH. Total hysterectomies accounted for 79.8% of hysterectomies, 16.1% were subtotal, and 4% were radical or modified radical hysterectomies. There were no significant differences between patients who had a subtotal and those that had a total hysterectomy for BMI, age, LOS, length of surgery, number of infections, or number of complications. The patients differed only in terms of parity, in that those who underwent a total hysterectomy had more children (2.12 versus 1.66, p = 0.026).

A concurrent procedure was performed in 26.6% of patients. This included biopsies (10.5%), reparative surgery (5.9%), procedures to establish urinary continence (3.5%), appendectomies (1.9%), and surgery to manage intra-operative events (2.2%). Table 2 outlines the indications for surgery overall and by type of hysterectomy. There were 526 indications listed for the 372 patients, as up to three reasons could be cited. For 245 of the women (65.9%), only one reason was identified, while 100 women (26.9%) had two reasons and an additional 27 (7.3%) had three reasons listed. Dysfunctional or abnormal uterine bleeding was the most common indication, at 26.4% of the sample. However, this indication accounted for 52.5% of the vaginal hysterectomies, while another 25.4% of the vaginal hysterectomies were for pelvic organ prolapse of stress incontinence. Significance testing of the indications by type of surgery was not carried out due to the large number of cells with a frequency of five or less.

**Table 2:** Indications for surgery by type of hysterectomy.

Indication	Abdominal	Vaginal	Lap-Assisted Vaginal	Vaginal Converted to Abdominal	Total (Row Percent)	Percent of Overall Total
Dysfunctional or Abnormal Uterine Bleeding	95 (68.3)	31 (22.3)	7 (5.1)	6 (4.3)	139 (100)	26.4%
Leiomyomas	80 (95.2)	3 (3.6)	0	1 (1.2)	84 (100)	16.0%
Adnexal or Pelvic Mass, Ovarian Neoplasm or Cyst	60 (100)	0	0	0	60 (100)	11.4%
Endometrial, Ovarian or Cervical Cancer	54 (93.1)	3 (5.2)	0	1 (1.7)	58 (100)	11.0%
Chronic Pelvic Pain, Severe Menstrual Related Mood Disorder or Dysmenorrhea	38 (67.9)	4 (7.1)	13 (23.2)	1 (1.8)	56 (100)	10.6%
Endometrial Hyperplasia, Cervical Dysplasia, or Family or Personal History of Cancer	37 (78.7)	3 (6.4)	6 (12.8)	1 (2.1)	47 (100)	8.9%
Pelvic Organ Prolapse or Genuine Stress Incontinence	22 (55.0)	15 (37.5)	2 (5.0)	1 (2.5)	40 (100)	7.6%
Endometriosis or Adenomyosis	22 (81.5)	0	4 (14.8)	1 (3.7)	27 (100)	5.1%
Chronic Salpingitis, Oophoritis, Hydrosalpinx, Pyosalpinx, Post Menopausal Bleed or Other	15 (100)	0	0	0	15 (100)	2.9%
Total	423 (80.4)	59 (11.2)	32 (6.1)	12 (2.3)	526 (100)	100%

Values are given as N (% of row total), with the exception of the final column, which contains the percentage of the overall total. Note that up to three indications could be listed, resulting in 526 reasons for 372 patients.

Fifty-eight (15.6%) of the patients had a diagnosis of cancer pre-operatively, which rose to 76 (20.4%) post-operatively. The population with cancer was older, had higher BMIs, longer surgeries, and longer lengths of stay than those without cancer (Table 3).

**Table 3:** Characteristics of patients with and without cancer.

Characteristic	Cancer	N	Mean	Std. Deviation	p-value*
BMI	No	285	28.0	6.1	.042
	Yes	72	30.7	10.7	
Age in years	No	296	46.7	10.1	< .001
	Yes	76	55.9	13.9	
Length of Surgery in minutes	No	293	98.9	41.1	< .001
	Yes	76	125.7	58.4	
Length of Stay in days	No	296	4.3	3.4	.022
	Yes	76	6.2	7.0	

* p-values are based on the two-sample t-test

BMI was missing for 4 patients with cancer and 11 patients without cancer; length of surgery in minutes was missing for 3 patients without cancer.

Twenty-six patients visited the emergency room within 30 days of their discharge and an additional nineteen patients were readmitted to the hospital. Table 4 compares the characteristics of patients who were not readmitted to those who were seen in the ER or readmitted to the hospital.

**Table 4:** Characteristics of patients readmitted to the ER or hospital.

Characteristic	Readmission	N	Mean	Std. Deviation	p-value*
BMI	None	303	28.4	6.7	.007
	ER Only	25	27.1	6.9	
	Readmitted	18	33.6	13.4	
Age in years	None	316	48.9	11.5	.110
	ER Only	26	44.0	9.9	
	Readmitted	19	47.6	12.7	
Length of Stay in days	None	316	4.6	3.4	.007
	ER Only	26	3.8	1.5	
	Readmitted	19	7.7	13.4	

* p-values are based on one-way analysis of variance

Infections occurred in 15.3% of patients, including urinary tract infections (7.5%), incision site infections (5.6%) and pelvic infections (2.2%). Those who developed an infection had a higher mean BMI (p = 0.018), longer LOS (p = 0.018) and longer length of surgery (p = 0.036) than those who did not.

Four percent of patients had a repeat laparotomy or unplanned laparotomy (not including those for conversion of VH to AH). Other complications occurred in 24.5% of patients, the most common being excessive bleeding (11.3%) and post-operative ileus (5.4%). Other complications involving the bladder, bowel, pulmonary function, cardiac function or drug reactions occurred in less than 2% of patients respectively.

Table 5 contains the characteristics of the women by oophorectomy and hysterectomy type (excluding LAVH and VH converted to AH). Overall, 65% of women had both or last ovary removed, including 57% of the 257 premenopausal women and 84% of the 113 postmenopausal women (menopausal status was not documented in two patients). In women with dysfunctional uterine bleeding as the only indication, 35% had both or last ovary removed. In women with leiomyomas as the only indication, 71.4% had both or last ovary removed.

**Table 5:** Characteristics of Women By Oophorectomy and Hysterectomy Categories

Characteristic Mean (SD)	Oophorectomy		Hysterectomy	
	
	No Ovaries Removed n = 129	Both or Last Ovary Removed n = 243	Abdominal n = 275	Vaginal n = 52
Age in Years	44.3 (10.7)	50.8 (11.4)*	49.4 (11.5)	47.4 (11.9)
Body Mass Index	27.4 (5.3)	29.2 (8.2)*	29.2 (7.8)	25.8 (4.6)†
Length of Stay in Days	3.8 (1.7)	5.2 (5.2)*	5.2 (4.8)	3.0 (1.6)†
Length of Surgery in Minutes	109.3 (56.8)	101.8 (39.8)	106.3 (48.7)	84.7 (34.6)†

* Between-group differences significant at p < .05, 2-sample t-test

† Between-group differences significant at p < .01, 2-sample t-test

A comparison of the abdominal and vaginal approaches revealed no differences in terms of incidence of infection, readmission to the ER or hospital, incidence of excessive bleeding or complication rate.

LAVH and VH converted to AH were excluded from all regression analyses as they represented subgroups that were clinically different than routine AH and VH. Table 6 presents the results of the linear regression modeling for length of surgery and LOS. All variables with a significance level of p < .20 in the bivariate analyses were offered into the models. Predictors of length of surgery included higher BMI, younger age, higher parity, a higher number of concurrent procedures and an abdominal approach. These predictors account for 33.1% of the variation in length of surgery. Predictors of a longer LOS include an abdominal approach, older age and a higher number of concurrent procedures. Oophorectomy, which was significantly associated with LOS in the bivariate analyses, was not retained in the model since it was also associated with the abdominal approach, resulting in collinearity between the two variables. In order to normalize the distribution, the LOS regression model was developed without two outliers that had LOS of 45 and 62 days. The three predictors accounted for 19% of the variation in LOS. Post-hoc analyses (scatter plots of the residuals against the predicted values, influence diagnostics) were done to examine the model fitting and indicated that the fit was acceptable.

**Table 6:** Predictors of Length of Surgery and Length of Stay based on Linear Regression

**Length of Surgery in minutes (r^2 ^= .331)**	**Coefficient**	**p-value**
Constant	77.11	
BMI	1.16	< .001
Age in years	-0.55	.008
Parity	4.06	.009
Number of concurrent procedures	45.24	< .001
Vaginal approach (compared to abdominal)	-22.56	< .001
**Length of Stay in Days (r^2 ^= .189)**		
Constant	2.3	
Vaginal approach (compared to abdominal)	-1.7	< .001
Age in years	0.043	< .001
Number of concurrent procedures	1.2	< .001

Additional variables offered into the Length of Surgery model (but not selected) included menopausal status, number of indications, cancer as primary indication and oophorectomy.

Additional variables offered into the Length of Stay model included BMI, cancer as primary indication and oophorectomy.

Logistic regression for approach of hysterectomy indicated that a patient was 1.1 times more likely to have an AH for each one-point increase in BMI (p = 0.003), 47.6 times more likely to have an AH if she had a concurrent unilateral or bilateral oophorectomy (p < 0.001) and 1.7 times more likely to have a VH with each additional child (p < 0.001).

## Discussion

The majority of the patients were overweight (29.6%, BMI 25–29.9) or obese (36.6%, BMI ≥ 30). These numbers define a population whose obesity level is 21.8 percentage points above the national average and although there is no known average BMI for all hysterectomy patients in Canada for comparison, the high obesity rate at this centre may have contributed to the reliance on the abdominal approach. A patient was in fact eleven times more likely to have an AH for every 10-point increase in BMI. Although recent studies exclude BMI as a factor in determining the route of hysterectomy, it has been noted that obesity of the buttocks may interfere with the exposure necessary for a VH [5].

The general trend in determining the route of hysterectomy has been to challenge the validity of the exclusionary criteria for VH, such as nulliparity, larger uterine size, previous cesarean delivery, and pelvic laparotomy. These are no longer considered to be strong contraindications to a vaginal approach [5-11]. Yet the abdominal approach is still the most utilized approach at this facility, accounting for 78% of the hysterectomies. The general impression from this and other studies is that surgeon expertise, patient weight and the need for adnexal surgery may play the strongest roles in determining the ultimate route for hysterectomy [6-12]. The need for concurrent oophorectomy may also have been a contributing factor. Oophorectomies, while able to be performed vaginally in the majority of circumstances, were more likely to have been performed abdominally in this population due to issues of accessibility (size of patient).

The overall ratio of abdominal to vaginal (alone or in conjunction with laparoscopy) surgeries is 5.6:1 but when only considering those surgeries performed for indications other than cancer (cancer found pre or post operatively), the ratio reduces to 3.9:1. This is consistent with the fact that most malignant indications for surgery require an abdominal approach in order to ensure access to structures and to allow for staging procedures. Our data did not demonstrate a significant difference between AH and VH in terms of outcome variables such as the rate of infection or complication, however, the two day reduction in LOS for VH may have significant cost reduction potential [8,13]. In our study, less than 20% of the hysterectomies performed in 2001 were VH or LAVH. This is below the average rate of 32% across Canada for 1999–2000 [14]. The average length of stay for hysterectomy was 4.7 days, which is only slightly above the average Canadian value of 4.4 from 1999–2000 [14]. In light of this comparison, an effort to increase the proportion of hysterectomies performed using a vaginal approach would be in keeping with the Society of Obstetricians and Gynecologists of Canada clinical practice guidelines which recommend offering VH to all women where that approach is deemed feasible by the surgeon [15]. Recent reports [16] have demonstrated a marked improvement in the ratio of VH to AH with the adoption of guidelines that clearly determine the correct surgical approach based on vaginal access, mobility with the Valsalva maneuver and uterine size. The application of guidelines [17] such as these warrants careful consideration in centers where a mandate exists to increase the rate of VH.

The merit of performing a concurrent oophorectomy during hysterectomy continues to be debated for women not at high risk of developing ovarian cancer. Estimates regarding the number of prophylactic oophorectomies needed to prevent one case of ovarian cancer range from 200 [18] to 300 [19]. Benefits such as prevention of ovarian cancer and perhaps breast cancer have to be weighed against an instantaneous surgical menopause that may increase a woman's risk of ischemic heart disease and osteoporosis [18]. In addition, although not all women decide to take HRT after oophorectomy, those that do, have to additionally consider the risks and benefits associated with that treatment. The main outcome from a recent study that investigated women's attitudes towards oophorectomy as an adjunct to hysterectomy concluded that while over half the women expressed a desire to decline oophorectomy, the majority were not well informed as to the long-term consequences of either decision [20]. Few clear guidelines exist to aid either the physician or the patient in the decision making process, making it all the more important to ensure that the patient is adequately informed about the long and short term risks and benefits of all treatment options.

The limitations of this study include uneven distribution of patients in each treatment group and lack of randomization due to the nature of the retrospective chart review process. Furthermore, because the audit process relied entirely on chart documentation, information may have been missed or incorrect as a result of improper or absent documentation. The broad range of information collected also prevented the researchers from employing more rigorous definitions and verification of outcomes. The retrospective nature of the study precluded an evaluation of the decision making process leading to oophorectomy as well as the influence of pre-operative indications, uterine size, parity, previous c-section and concurrent oophorectomy on surgical approach. This would need to be addressed prospectively, by surveying the surgeons at the time that the decision was made.

## Conclusions

Both the patient and the health care system may benefit from the trend towards increased use of vaginal hysterectomies. However, the abdominal approach continues to dominate, likely related to patient size, surgeon preference and the need for adnexal surgery. The audit process proved to be an important method by which to assess trends associated with common surgical procedures. This study raises important questions about the relationship between patient characteristics, surgical approach and the indications for surgery, and a prospective approach, designed to address these questions more fully, is now indicated. Furthermore, in light of recent evidence [16] demonstrating the impact of a directed approach to affect the ratio of AH to VH, clear guidelines as provided by the Society of Pelvic Reconstructive Surgeons [17] should be considered to invariably increase the rate of VH. This study raises important questions about the relationship between patient characteristics, surgical approach and the indications for surgery, and a prospective approach, designed to address these questions more fully, is now indicated.

## Competing Interests

The author(s) declare that they have no competing interests.

## Authors' Contributions

AT performed data collection, participated in the study design and coordination and participated in drafting the manuscript. WH performed the statistical analysis and participated in drafting the manuscript. RHG conceived the study and participated in its design and coordination and participated in drafting the manuscript. All authors read and approved the final manuscript.

## Pre-publication history

The pre-publication history for this paper can be accessed here:


